# Myosin Binding Protein-C Slow Phosphorylation is Altered in Duchenne Dystrophy and Arthrogryposis Myopathy in Fast-Twitch Skeletal Muscles

**DOI:** 10.1038/srep13235

**Published:** 2015-08-19

**Authors:** Maegen A. Ackermann, Christopher W. Ward, Christina Gurnett, Aikaterini Kontrogianni-Konstantopoulos

**Affiliations:** 1 University of Maryland, School of Medicine, Department of Biochemistry and Molecular Biology, Baltimore, MD, USA; 2 University of Maryland, School of Nursing, Baltimore, MD, USA; 3 Washington University, School of Medicine, Department of Neurology, St. Louis, MO, USA

## Abstract

Myosin Binding Protein-C slow (sMyBP-C), encoded by *MYBPC1*, comprises a family of regulatory proteins of skeletal muscles that are phosphorylated by PKA and PKC. *MYBPC1* missense mutations are linked to the development of Distal Arthrogryposis-1 (DA-1). Although structure-function details for this myopathy are evolving, function is undoubtedly driven by sequence variations and post-translational modifications in sMyBP-C. Herein, we examined the phosphorylation profile of sMyBP-C in mouse and human fast-twitch skeletal muscles. We used Flexor Digitorum Brevis (FDB) isolated from young (~2-months old) and old (~14-months old) wild type and *mdx* mice, and human Abductor Hallucis (AH) and gastrocnemious muscles carrying the DA-1 mutations. Our results indicate both constitutive and differential phosphorylation of sMyBP-C in aged and diseased muscles. We report a 7–35% reduction in the phosphorylation levels of select sites in old wild type and young or old *mdx* FDB mouse muscles, compared to young wild type tissue. Similarly, we observe a 30–70% decrease in the phosphorylation levels of all PKA and PKC phospho-sites in the DA-1 AH, but not gastrocnemius, muscle. Overall, our studies show that the phosphorylation pattern of sMyBP-C is differentially regulated in response to age and disease, suggesting that phosphorylation plays important roles in these processes.

Myosin Binding Protein-C (MyBP-C) is a family of accessory proteins of striated muscles that contributes to thick filament assembly and stabilization, and modulates contraction by regulating the formation of actomyosin cross-bridges^1^,^2^,^3^,^4^,^5^,^6^,^7^,^8^. The slow (s) skeletal isoform of MyBP-C, which is encoded by the *MYBPC1* gene, is comprised of seven immunoglobulin (Ig) and three fibronectin-III (Fn-III) domains. The first Ig domain, C1, is flanked by a short stretch of amino acids (~50 aa) enriched in proline (Pro) and alanine (Ala) residues, termed Pro/Ala rich motif, and a conserved linker region (~100 aa), noted the M-motif. Differing from the cardiac and fast skeletal homologues, sMyBP-C consists of a heterogeneous family of proteins (126–131.5 kDa) that result from extensive exon shuffling^9^,^10^. The human transcriptome contains fourteen sMyBP-C transcripts, which encode fourteen distinct variants, differing by small segments of amino acids within the Pro/Ala rich motif, the M-motif, the Ig domain C7, and the extreme COOH-terminus^10^. To date, five full-length sMyBP-C variants have been identified in the mouse transcriptome, however it is likely that the known human variants correspond to unidentified mouse variants. Interestingly, the sMyBP-C variants are co-expressed in both slow and fast twitch skeletal muscles where they may exhibit distinct topographies and functions^1^,^2^,^9^.

MyBP-C slow is phosphorylated at four residues within the Pro/Ala rich region and the M-motif at its NH_2_-terminus^11^. These four sites (mSer-59/hSer-61, mSer-62/hThr-64, mThr-84/hSer-85, and mSer-204/hSer-206) are conserved between mouse (m) and human (h) sMyBP-C and are phosphorylated by PKA and/or PKC^11^. Specifically, mSer-59/hSer-61 and mSer-62/hThr-64 are targeted by PKA, mThr-84/hSer-85 is phosphorylated by PKC, and mSer-204/hSer-206 is a substrate of both PKA and PKC. Of the four phosphorylation sites, mSer-62/hThr-64 and mThr-84/hSer-85 are constitutively expressed in all known mouse and human variants (Fig. 1, grey highlighted residues). Contrary to this, mSer-59/hSer-61 and mSer-204/hSer-206 are encoded by exons that are alternatively spliced, and are thus present only in select variants (Fig. 1, purple and green highlighted residues, respectively). Accordingly, mSer-59/hSer-61, present within the Pro/Ala rich motif, is expressed only in h-variant (v) 1, h-v2, and m-isoform3, while mSer-204/hSer-206, present within the M-motif, is expressed in all variants with the exception of h-v8. Therefore, alternative splicing and phosphorylation may regulate the activities of the different sMyBP-C variants.

Genomic linkage analysis has demonstrated that mutations in *MYBPC1* are causally linked to the development of distal arthrogryposis type-1 (DA-1)^12^. DA-1 is the most common among the ~10 forms of distal arthrogryposis myopathy that have been described to date affecting approximately 1 in 10,000 individuals^13^,^14^. Two dominant missense mutations, W236R and Y856H, have been identified, which are located in the M-motif and C8 domain, respectively. Although the DA-1 causing mutations are present in constitutively expressed exons and thus contained in all slow variants expressed in skeletal muscles, DA-1 patients have contractures that are limited to the distal muscles of the hands and feet, including clubfoot, vertical talus, camptodactyly, overriding fingers and ulnar deviation of fingers with no additional anomalies^12^. The congenital nature of the DA-1 contractures has been attributed to the early embryonic expression of sMyBP-C, which precedes that of *MYBPC2* encoding the fast skeletal isoform^15^. Importantly, the presence of the DA-1 mutations abolishes the proteins’ ability to interact with actin and myosin and regulate the formation of cross-bridges *in vitro*^2^. However, the effects of either of the DA-1 mutations on the phosphorylation profile of sMyBP-C are still unknown. Along the same lines, earlier studies have demonstrated that loss of dystrophin in Duchenne Muscular Dystrophy (DMD) leads to a number of adaptive and maladaptive responses, including alterations in global protein phosphorylation^16^,^17^. As in DA-1 though, the phosphorylation profile of sMyBP-C has yet to be described in DMD.

Using a panel of phospho-specific antibodies in conjunction with standard and phosphate affinity SDS-PAGE, we examined the phosphorylation profile of sMyBP-C in different mouse and human skeletal muscles. Specifically, we compared the phosphorylation profile of sMyBP-C in young (~2 months old) and old (~14 months old) fast-twitch Flexor Digitorum Brevis (FDB) muscles obtained from wild type and *mdx* mice, and human Abductor Hallucis (AH) and gastrocnemius fast-twitch muscles carrying the Y856H and W236R mutations, respectively. Our studies demonstrate that while overall phosphorylation is only moderately decreased at select sites in young and old wild type and *mdx* muscles, there are qualitative and quantitative differences in individual phosphorylation events as a result of age and/or dystrophy. Moreover, the phosphorylation profile of sMyBP-C is significantly altered in human AH muscle, which is severely affected in DA-1, but not in gastrocnemius muscle, which shows no phenotypic manifestation of the disease. Collectively, our studies are the first to demonstrate that the phosphorylation pattern of sMyBP-C is differentially regulated in response to age and disease.

## Results

### Characterization of the phosphorylation profile of sMyBP-C in the mouse fast-twitch FDB muscle in health and dystrophy

Our recent studies have demonstrated that sMyBP-C encompasses a family of phosphoproteins with mSer-59 and mSer-62 phosphorylated by PKA, mThr-84 phosphorylated by PKC, and mSer-204 phosphorylated by both PKA and PKC^11^. To study the phosphorylation profile of sMyBP-C in fast-twitch skeletal muscles in response to different (patho)physiological stressors, such as aging and disease, we selected the mouse FDB muscle because of the abundant expression of several sMyBP-C variants^9^,^10^. We produced lysates from young (~2 months) and old (~14 months) wild type and *mdx* FDB muscles, which were analyzed by conventional and phosphate-affinity gel electrophoresis and subsequent immunoblotting. Moreover, we used a panel of antibodies including a sMyBP-C antibody that recognizes constitutively expressed epitopes in the C5 domain (α-pan-sMyBP-C), and four phospho-specific antibodies recognizing all known phosphorylation sites (α-sMyBP-C mSer59P, α-sMyBP-C mSer62P, α-sMyBP-C mThr84P, and α-sMyBP-C mSer204P). It is important to note that due to the presence of multiple sMyBP-C variants with similar molecular weights (126–131.5 kDa)^10^, the immunoreactive bands detected in our phosphate-affinity blots may correspond to unique variants phosphorylated to differing extents, differentially phosphorylated forms of the same variant, or a combination of both. Consequently, we refrain from denoting those as hyper- or hypo-phosphorylated species and instead refer to them according to their electrophoretic mobility as low, intermediate, or high. Moreover, we consider young wild type FDB tissue as reference, and report qualitative and quantitative differences in the other conditions that deviate from it.

**Figure 2:**
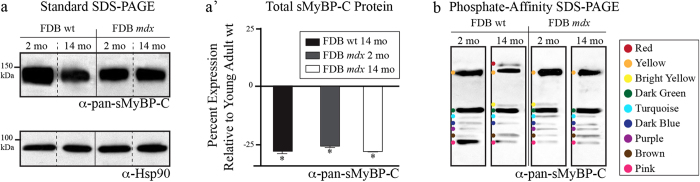
Evaluation of the phosphorylation profile of sMyBP-C in young and old wild type and *mdx* FDB muscles. (**a**) Standard SDS-PAGE western blot analysis of protein lysates prepared from young (~2 months) and old (~14 months) wild type and *mdx* FDB muscles. Lysates were probed with a α-pan-sMyBP-C (top panel) recognizing all sMyBP-C variants, and α-Hsp90 (bottom panel) to ensure equal loading. (**a’**) Percent expression of total sMyBP-C in young and old wild type and *mdx* FDB muscles, after normalization to the levels of Hsp90, and relative to the percent expression of sMyBP-C in young wild type tissue, which was set as baseline. Significance was calculated via student’s t-test (p < 0.01). The star (*) symbol denotes significance compared to young wild type. (**b**) Separation of the same protein lysates used in panel (**a**) via phosphate affinity SDS-PAGE followed by immunoprobing with the α-pan-sMyBP-C antibody. Colored dots represent immunoreactive bands of low (red and yellow dots), intermediate (bright yellow, dark green, turquoise, dark blue, purple, and brown dots), and high (pink dot) mobility. Four animals (n = 4) were used per group.

Using standard SDS-PAGE immunoblotting, we examined the expression levels of sMyBP-C in the different FDB muscles described above. We observed a consistent and significant reduction (~25%) in the total levels of sMyBP-C in aged wild type and young or aged *mdx* FDB muscles, compared to young wild type tissue (Fig. 2a,a’). Analysis of the phosphorylation profile of sMyBP-C in young wild type FDB muscle using the α-pan-sMyBP-C antibody and phosphate-affinity SDS-PAGE indicated the presence of seven distinct forms of different electrophoretic mobility (Fig. 2b). For ease of presentation, we use colored dots to denote the observed immunoreactive bands. We detected one low mobility band (yellow dot), five intermediate mobility bands (dark green, turquoise, dark blue, purple, and brown dots), and one high mobility band (pink dot). Aged wild type FDB muscle expresses six of the seven sMyBP-C species present in the young wild type tissue, in addition to two new forms of low (red dot) and intermediate (bright yellow dot) mobility, thus containing a total of eight sMyBP-C phospho-species (Fig. 2b). Young and aged *mdx* FDB muscles also express the same seven major forms as young wild type FDB muscle, in addition to an intermediate mobility species (bright yellow dot) that is also present in aged wild type FDB muscle (Fig. 2b).

### Evaluation of the phosphorylation levels of mSer59, mSer62, mThr84, and mSer204 of sMyBP-C in mouse FDB muscle in health and dystrophy

Using standard SDS-PAGE along with our panel of phospho-specific antibodies (Ackermann *et al.* 2014; Companion paper), we examined the phosphorylation levels of each known phospho-site within the NH_2_-terminus of sMyBP-C in young and aged wild type and *mdx* FDB muscles. As above, the young wild type FDB muscle was used as the basis for qualitative and quantitative comparisons.

**Figure 3:**
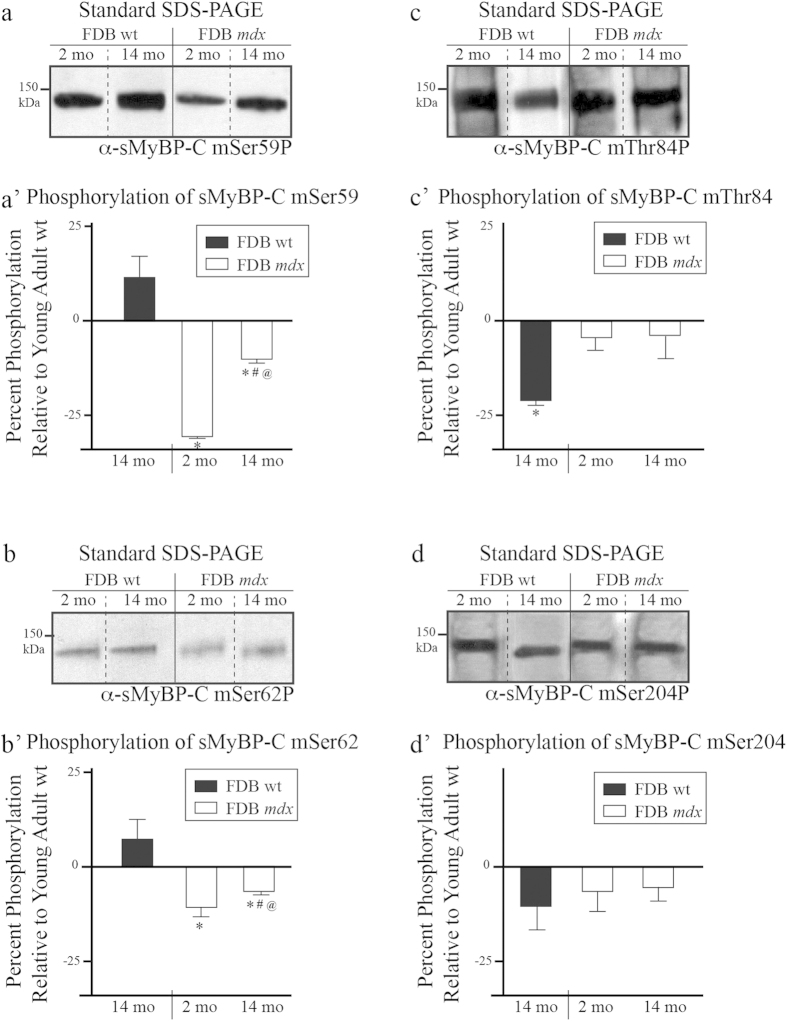
Examination of the phosphorylation levels of mSer59, mSer62, mThr84, and mSer204 of sMyBP-C in fast-twitch FDB muscles of young and old wild type and *mdx* mice. Western blot analyses were performed using standard SDS-PAGE and lysates prepared from young (~2 months) and old (~14 months) wild type and *mdx* FDB muscles. Samples were probed with α-sMyBP-C mSer59P (**a**), α-sMyBP-C mSer62P (**b**), α-sMyBP-C mThr84P (**c**), and α-sMyBP-C mSer204P (**d**) recognizing the four known phospho-sites of sMyBP-C. The levels of each phosphorylated residue, mSer59 (**a’**), mSer62 (**b’**), mThr84 (**c’**), and mSer204 (**d’**) in the different FDB muscles were calculated as percent expression relative to those in young wild type tissue, as described in Fig. 2. Four animals (n = 4) were used per group. Significance was calculated via student’s t-test (p < 0.01). The symbols, *, #, and @ denote significance compared to young wild type, young *mdx*, and old wild type, respectively.

Analysis of lysates probed with the mSer59P, mSer62P, mThr84P, or mSer204P antibodies indicated that young and aged, wild type and *mdx* FDB muscles contain sMyBP-C variants that are phosphorylated at each of the corresponding sites (Fig. 3a–d). Use of the mSer59P antibody demonstrated that the expression levels of phospho (p) mSer59-containing variants remain unaltered in aged wild type FDB muscle, compared to young wild type tissue; however, they decrease dramatically in young *mdx* (~34%) and moderately in old *mdx* (~11%) FDB muscles (Fig. 3a’). Similar quantitation of the amounts of p-mSer62-containing proteins showed that while aged wild type FDB contains similar levels to young wild type tissue, young and old *mdx* FDB express reduced levels (~12% and ~8%, respectively) (Fig. 3b’). Contrary to this, evaluation of the expression levels of p-mThr84-containing variants revealed that compared to young wild type tissue, young and aged *mdx* FDB contain similar levels, while old wild type FDB exhibits reduced amounts by ~21% (Fig. 3c’). Lastly, quantitation of the levels of p-mSer204-containing proteins showed that they remain unaltered in old wild type and young or old *mdx* FDB, compared to young wild type tissue (Fig. 3d’).

### Identification of the phosphorylation events present in individual sMyBP-C forms in young and aged healthy and diseased FDB muscles

**Figure 4:**
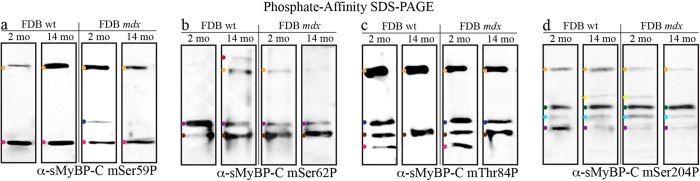
Identification of distinct phosphorylation events present in individual sMyBP-C forms in fast-twitch FDB muscles of young and old wild type and *mdx* mice. Western blot analysis using phosphate affinity SDS-PAGE blots of protein lysates obtained from young (~2 months) and old (~14 months) wild type and *mdx* FDB muscles were probed with α-sMyBP-C mSer59P (**a**), α-sMyBP-C mSer62P (**b**), α-sMyBP-C mThr84P (**c**), and α-sMyBP-C mSer204P (**d**). Colored dots denote low, intermediate, and high mobility sMyBP-C species identified with each antibody, and correspond to those shown in Fig. 2. Four animals (n = 4) were used per group.

**Figure 5:**
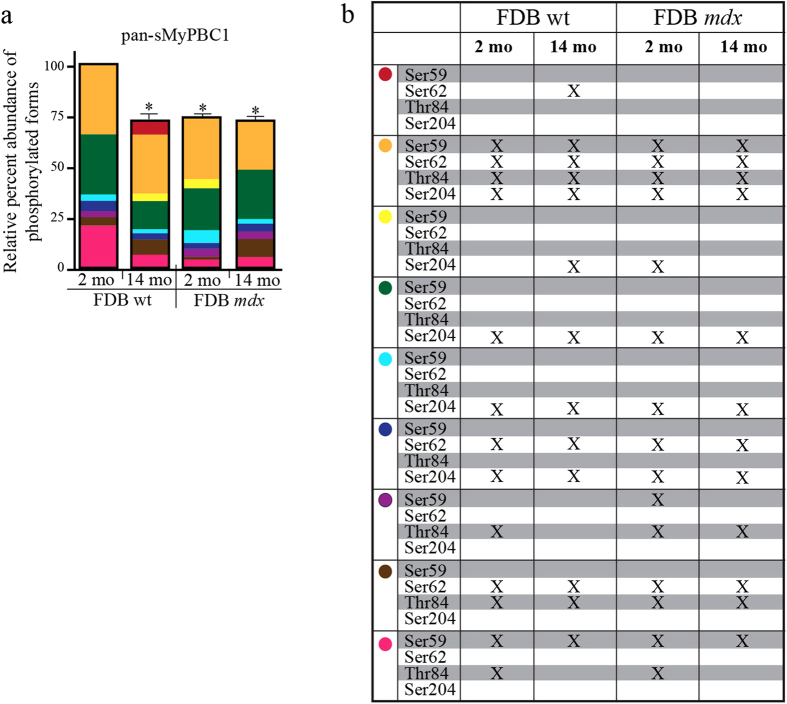
Relative abundance and phosphorylation profile of distinct sMyBP-C forms in fast-twitch FDB muscles of young and old wild type and *mdx* mice. (**a**) The relative abundance of each immunoreactive band detected with the α-pan-sMyBP-C antibody following separation of protein lysates by phosphate affinity SDS-PAGE (Fig. 2b) was calculated as percent expression of the total protein content within each sample. Significance is as described in Fig. 2a’. (**b**) The phosphorylation events detected in each sMyBP-C form across the different FDB muscle groups were identified by comparative evaluation of the phosphate affinity SDS-PAGE immunoblots shown in Fig. 4. Notably, colored dots correspond to sMyBP-C immunoreactive bands with specific electrophoretic mobility, as determined by phosphate affinity SDS-PAGE, and thus may represent different (i.e. bands denoted with purple and pink dots) or the same (bands denoted with red, yellow, bright yellow, dark green, turquoise, dark blue, and white dots) phospho-species among FDB samples.

To identify the phosphorylation events present within the individual sMyBP-C phospho-species detected with our α-pan-sMyBP-C antibody (Fig. 2b), we used phosphate affinity SDS-PAGE and our panel of phospho-specific antibodies (Figs 4 and 5).

Protein lysates prepared from young wild type FDB muscle probed with the mSer59P antibody contain two main species, one of low mobility (yellow dot) and one of high mobility (pink dot) (Fig. 4a). These are the only two phospho-species present in aged wild type and *mdx* FDB muscles, too, while young *mdx* FDB also expresses an intermediate mobility band (dark blue dot) (Fig. 4a).

Use of the mSer62P antibody revealed the presence of common and unique phospho-species compared to the mSer59P antibody. Two intermediate mobility species (purple and brown dots) were detected in young wild type FDB muscle (Fig. 4b). These are also present in aged wild type and young and aged *mdx* FDB muscles, albeit in differing amounts (Fig. 4b). Aged wild type FDB also expresses two low mobility p-mSer62-containing forms (red and yellow dots). Of those, the low mobility band denoted by the yellow dot is present in young *mdx* FDB samples, too (Fig. 4b).

Similar analysis using the mThr84P antibody indicated the presence of four major species in young wild type and *mdx* FDB muscles (Fig. 4c, yellow, dark blue, brown, and pink dots). Notably, p-mSer59 and/or p-mSer62 antibodies detect these species, too (Fig. 4c). Examination of aged wild type and *mdx* tissues indicated a similar expression profile of p-mThr84-containing species, compared to young wild type and *mdx* FDB muscles, with the following exceptions: aged wild type tissue lacks a high (pink dot) and an intermediate (dark blue dot) mobility species, while aged *mdx* tissue only lacks the high mobility species (pink dot) (Fig. 4c).

Lastly, evaluation of the expression profile of p-mSer204-containing proteins indicated the presence of four main species in young wild type FDB muscle, one of low mobility (yellow dot) and three of intermediate mobility (dark green, turquoise, and purple dots) (Fig. 4d). While the low (yellow dot) and intermediate (purple dot) mobility species are detected by other phospho-antibodies, the remaining two species (dark green and turquoise dots) are unique to mSer204P antibody. Similar to young wild type, aged wild type and young and aged *mdx* FDB muscles express the same four p-mSer204-containing forms (Fig. 4d), with the aged wild type and young *mdx* tissues containing an additional form of intermediate mobility (bright yellow dot) (Fig. 4d).

### Examination of the phosphorylation profile of sMyBP-C in human abductor hallucis and gastrocnemius muscles from DA-1 patients

**Figure 6:**
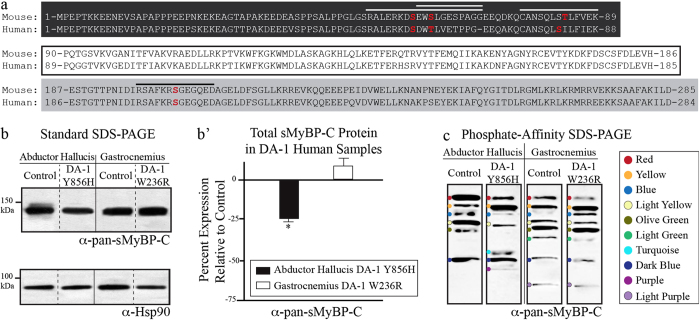
Examination of the phosphorylation profile of sMyBP-C in Abductor Hallucis (AH) and gastrocnemius muscles from human patients carrying the DA-1 mutations. (**a**) Alignment of sMyBP-C NH_2_-termini between mouse isoform 3 and human v1. Dark grey, white, and light grey boxes mark the Pro/Ala rich motif, domain C1, and the M-motif, respectively. The peptide sequences that were used for the generation of the four phospho-antibodies are marked with a line; notably, the sequence similarity and identity for each antigenic peptide between mouse and human are 100% and 90% for the Ser59 peptide, 82% and 45% for the Ser62 peptide, 100% and 85% for the Thr84 peptide, and 100% and 100% for the Ser204 peptide, respectively. (**b**) Standard SDS-PAGE western blot analysis of protein lysates prepared from human biopsies of AH and gastrocnemius muscles carrying the Y856H and W236R DA-1 mutations, respectively, or control muscles; for details on the DA-1 and control muscles please see the Materials and Methods section. Lysates were probed with α-pan-sMyBP-C (top panel) and α-Hsp90 (bottom panel). (**b’**) Percent expression of total sMyBP-C in DA-1 AH and gastrocnemius muscles after normalization to the levels of Hsp90, and relative to the percent expression of sMyBP-C in matching controls. Significance was calculated via student’s t-test (p < 0.01). The star (*) symbol denotes significance compared to controls. (**c**) Separation via phosphate affinity SDS-PAGE of the same protein lysates used in panel (**b**) followed by probing with the α-pan-sMyBP-C antibody. Colored dots represent immunoreactive bands of low (red, yellow, and blue dots), intermediate (light yellow, olive green, light green, turquoise, dark blue, and purple dots), and high (light purple dot) mobility.

Two missense mutations, W236R and Y856H, in human *MYBPC1* have been causally linked to the development of distal arthrogryposis type-1 (DA-1), affecting the distal muscles of the hands and feet^12^. W236R and Y856H are located within the M-motif and C8 domain, respectively. Both mutations reside in constitutively expressed exons, and are therefore present within all known sMyBP-C variants. To study the effects of each of these mutations on the phosphorylation profile of sMyBP-C, we used human biopsies of the abductor hallucis (AH) and gastrocnemius muscles carrying the Y856H and W236R mutations, respectively; AH and gastrocnemius muscles that did not contain the DA-1 mutations served as controls. We generated protein lysates from the DA-1 and control muscles and analyzed them by conventional or phosphate affinity gel electrophoresis and subsequent immunoblotting with the α-pan-sMyBP-C and the four phospho-specific antibodies. As a result of the high homology of the four phospho-sites between mouse and human (Fig. 6a), our panel of phospho-antibodies efficiently recognizes the human sMyBP-C proteins, too. Similar to our results with FDB (above) and soleus (Ackermann *et al.*, 2014; Companion paper), the immunoreactive bands detected in the phosphate affinity blots may correspond to different variants phosphorylated to variable extents, differentially phosphorylated forms of the same variant, or a combination of both. We therefore also refer to the immunoreactive bands detected in the human DA-1 and control muscles as low, intermediate, or high mobility, and we further correlate them with the respective bands detected in mouse FDB (this study) and soleus (Ackermann *et al.*, 2014; Companion paper) muscles, when applicable.

Using standard SDS-PAGE immunoblotting, we observed a significant reduction (~25%) of total sMyBP-C in DA-1 AH muscle carrying the Y856H mutation, compared to control tissue (Fig. 6b,b’). On the contrary, the sMyBP-C levels are unaltered in DA-1 gastrocnemius muscle containing the W236R mutation, compared to control muscle (Fig. 6b,b’). Examination of the phosphorylation profile of sMyBP-C in control AH muscle using the α-pan-sMyBP-C antibody and phosphate-affinity SDS-PAGE revealed six different species, three of low mobility (red, yellow, and blue dots) and three of intermediate mobility (light yellow, olive green, and dark blue dots) (Fig. 6c). DA-1 AH muscle expresses five of these species and two additional species of intermediate (turquoise dot) and high (purple dot) mobility. Similar examination of the phosphorylation profile of sMyBP-C in control gastrocnemius muscle showed eight different species, three of low (red, yellow, and blue dots), four of intermediate (light yellow, olive green, light green, and dark blue dots) and one of high (light purple dot) mobility. Contrary to DA-1 AH muscle, the phosphorylation profile of sMyBP-C is not altered in DA-1 gastrocnemius muscle carrying the W236R mutation, compared to control muscle.

### Evaluation of the phosphorylation levels of hSer61, hThr64, hSer85, and hSer206 of sMyBP-C in human abductor hallucis and gastrocnemius muscles from DA-1 patients

Using standard SDS-PAGE along with our panel of phospho-specific antibodies, we examined the phosphorylation levels of each phospho-site within the NH_2_-terminus of sMyBP-C in AH and gastrocnemius muscles carrying the Y856H and W236R mutations, respectively. As above, AH and gastrocnemius muscles served as controls, and were used as the basis for qualitative and quantitative comparisons.

**Figure 7:**
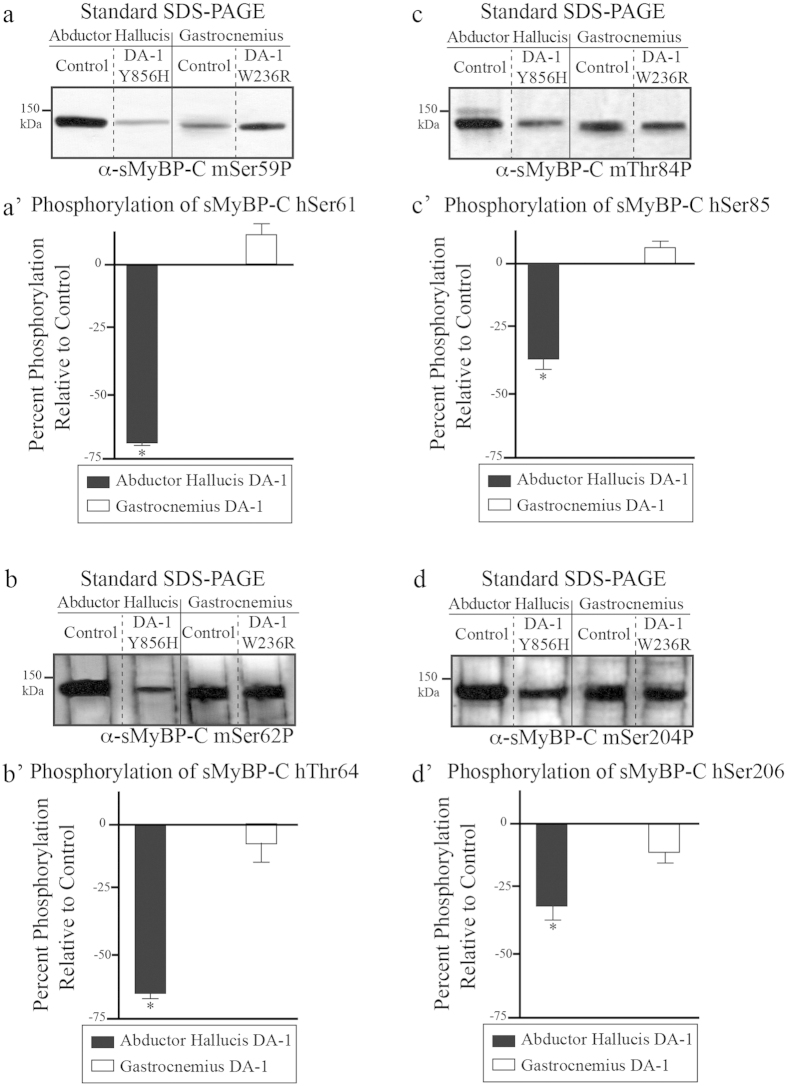
Examination of the phosphorylation levels of hSer61, hThr64, hSer85, and hSer206 of sMyBP-C in AH and gastrocnemius muscles from human patients carrying the DA-1 mutations. Western blot analyses were performed using standard SDS-PAGE of lysates prepared from human biopsies of AH and gastrocnemius muscles carrying the Y856H and W236R DA-1 mutations, respectively, and matched control tissues. Samples were probed with α-sMyBP-C mSer59P (**a**), α-sMyBP-C mSer62P (**b**), α-sMyBP-C mThr84P (**c**), and α-sMyBP-C mSer204P (**d**) recognizing the four known phospho-sites of sMyBP-C in humans, too. The levels of each phosphorylated residue, hSer61 (**a’**), hThr64 (**b’**), hSer85 (**c’**), and hSer206 (**d’**) in the DA-1 samples were calculated as percent expression relative to those in matching control tissues, as described in Fig. 6. Significance was calculated via student’s t-test (p < 0.01). The star (*) symbol denotes significance compared to controls.

Analysis of lysates probed with the mSer59P, mSer62P, mThr84P, and mSer204P antibodies indicated that control and DA-1 AH and gastrocnemius muscles express sMyBP-C variants that are phosphorylated at each of the four known sites (Fig. 7a–d). Interestingly though, the expression levels of variants phosphorylated at hSer61, hThr64, hSer85 or hSer206 are dramatically decreased by ~70%, ~65%, ~37% and ~32%, respectively, in DA-1 AH muscle carrying the Y856H mutation, compared to control tissue (Fig. 7a’–d’). On the contrary, DA-1 gastrocnemius muscle carrying the W236R mutation shows no significant changes in the phosphorylation levels of the four sites, compared to control (Fig. 7a’–d’).

### Examination of the phosphorylation events present in individual sMyBP-C forms in human abductor hallucis and gastrocnemius muscles from DA-1 patients

**Figure 8:**
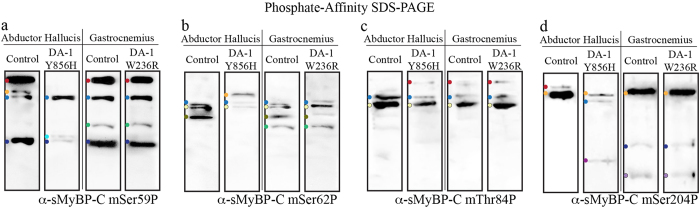
Identification of distinct phosphorylation events present in individual sMyBP-C forms in AH and gastrocnemius muscles from human patients carrying the DA-1 mutations. Western blot analysis using phosphate affinity SDS-PAGE blots of protein lysates obtained from human biopsies of AH and gastrocnemius muscles carrying the Y856H and W236R DA-1 mutations, respectively, and control muscles were probed with α-sMyBP-C mSer59P (**a**), α-sMyBP-C mSer62P (**b**), α-sMyBP-C mThr84P (**c**), and α-sMyBP-C mSer204P (**d**). Colored dots denote low, intermediate, and high mobility sMyBP-C species identified with each antibody, and correspond to those shown in Fig. 6.

We next examined the phosphorylation events present within the individual sMyBP-C species detected with our α-pan-sMyBP-C antibody (Fig. 6b) using phosphate-affinity SDS-PAGE and our panel of phospho-specific antibodies (Fig. 8). Control AH muscle expresses four main p-hSer61-containing species, three of low (red, yellow, and blue dots) and one of intermediate (dark blue dot) mobility (Fig. 8a). DA-1 AH muscle expresses the same low (blue dot) and intermediate (dark blue dot) mobility forms and an additional species of intermediate mobility (turquoise dot) (Fig. 8a). Contrary to this, similar analysis of control and DA-1 gastrocnemius muscles indicated that they express the same four main species, two of low mobility (red and blue dots) and two of intermediate mobility (light green and dark blue dots).

Three distinct p-hThr64-containing species of low (blue dot) and intermediate (light yellow and olive green dots) mobility were observed in control AH muscle (Fig. 8b). Two of them, including the low (blue dot) and intermediate (light yellow dot) mobility species are also present in DA-1 AH muscle, which also expresses an additional low mobility species (yellow dot) (Fig. 8b). Similar to the mSer59P antibody, the mSer62P antibody revealed a matching phosphorylation profile of sMyBP-C in control and DA-1 gastrocnemius samples, which express a low (blue dot) and three intermediate (light yellow, olive green, and light green dots) mobility species (Fig. 8b).

Moreover, control AH contains two major p-hSer85-containing species of low mobility (blue and light yellow dots), which are also present in DA-1 AH muscle in addition to an extra low mobility form (red dot) (Fig. 8c). As above, examination of control and DA-1 gastrocnemius muscles indicated an identical expression profile of p-hSer85-containing species, including three low mobility forms (red, blue, and light yellow dots) (Fig. 8c).

Lastly, evaluation of the expression profile of p-hSer206-containing proteins indicated the presence of two main species in control AH muscle, both of low mobility (red and yellow dots) (Fig. 8d). DA-1 AH tissue also expresses the low mobility species denoted by the yellow dot, along with additional low (blue dot) and intermediate (purple dot) mobility forms (Fig. 8d). Control gastrocnemius muscle contains three main species, of low (yellow dot), intermediate (dark blue dot), and high (light purple dot) mobility, all of which are shared with DA-1 gastrocnemius muscle (Fig. 8d).

## Discussion

### The phosphorylation profile of sMyBP-C in fast-twitch FDB muscle in health and dystrophy

To complement our assessment of the phosphorylation profile of the slow isoform of MyBP-C in slow-twitch soleus muscle (Ackermann *et al.* ’14; Companion paper), we herein determine its phosphorylation profile in fast-twitch FDB muscle under different (patho)physiological conditions. To this end, we used standard and phosphate-affinity gel electrophoresis in combination with a panel of phospho-specific antibodies to examine the phosphorylation profile of sMyBP-C in healthy and dystrophic FDB muscles of mouse origin at early and late adulthood. Interestingly, both age and dystrophy result in a significant decrease (~25%) in the total levels of sMyBP-C (Fig. 5a). This finding is in contradiction to our previous report showing no apparent differences in total sMyBP-C protein between 4 months old wild type and *mdx* FDB^11^. Underlying this contradiction is likely the temporal progression of disease pathology. In the *mdx* mouse, the pathological process is evident from birth but dramatically accelerates post-weaning, peaking at ~6 weeks and resolving to a stable slow progression after ~20 weeks of age. The evolution of disease progression is reflected in muscle function^18^,^19^,^20^,^21^, and likely underscores the differential effect we observe on protein expression in 2 months old mice herein versus the 4 months old mice interrogated in our previous report^11^. Notably, we also observed a significant reduction in the overall phosphorylation of residues mSer59 and mSer62, both targeted by PKA. This is consistent with previous findings indicating that the enzymatic activity of PKA is significantly reduced (~24%) in dystrophic muscles^22^, which is likely due to mislocalization of A Kinase Anchoring Proteins (AKAP), which are scaffolding proteins that modulate the proper targeting of the regulatory subunit of PKA^23^. Moreover, the presence and relative abundance of individual phosphorylation events or combinations thereof vary considerably in response to age or dystrophy (Fig. 5a,b). Our studies therefore clearly demonstrate that the phosphorylation profile of sMyBP-C in fast-twitch FDB muscle is altered in response to different (patho)physiological stressors.

To comprehensively present our findings shown in Figs 2, 3, 4, we analyzed the relative abundance (Fig. 5a) and phosphorylation profile (Fig. 5b) of the individual sMyBP-C species expressed within and among the four FDB muscles examined. Of the seven unique sMyBP-C forms expressed in young wild type FDB muscle, a low mobility species (yellow dot) is the most abundant one (~35%) and is phosphorylated at all four known residues. Of similarly high abundance (~30%) is an intermediate mobility species (dark green dot), which is singly phosphorylated at mSer204, while a high mobility species (pink dot) also displays a relatively high expression (~20%), and is phosphorylated at mSer59 and mThr84. The remaining sMyBP-C forms are of substantially lower abundance (~3–5%), and are either doubly or singly phosphorylated. Notably, young wild type FDB and soleus muscles express many of the same mobility sMyBP-C species (yellow, dark green, turquoise, dark blue, and pink dots), albeit to different amounts and occasionally of distinct phosphorylation profiles (Ackermann *et al.*, 2014; Companion paper, Fig. 6).

The molecular complexity of sMyBP-C may explain two paradoxical observations that we made in FDB muscle lysates analyzed by phosphate-affinity SDS-PAGE. sMyBP-C species containing one phosphorylation event (e.g. bands denoted with red and bright yellow dots) may migrate slower compared to species containing four phosphorylation events (e.g. band marked with a yellow dot). Moreover, sMyBP-C species exhibiting similar electrophoretic mobility among different FDB muscles may contain different numbers of phosphorylation events (e.g. bands denoted with purple and pink dots). Two possibilities may explain these findings: i. singly or doubly phosphorylated species may correspond to higher molecular weight variants (126–131.5 kDa), or ii. lower molecular weight species may contain multiple (known and potentially unknown) phosphorylation events. Unlike the majority of the sMyBP-C species expressed in soleus muscle that are phosphorylated at multiple, if not all, known sites (Ackermann *et al.* 2014; Companion paper, Fig. 6), many of the forms expressed in FDB muscle are singly or doubly phosphorylated (Fig. 5b). Given that single phosphorylation events were detected at any of the four known sites, it becomes apparent that these can happen independently. Interestingly, three sMyBP-C species (bright yellow, dark green, and turquoise dots) are singly phosphorylated at mSer204, yet they exhibit distinct electrophoretic mobilities. We speculate that these correspond to different sMyBP-C variants of distinct molecular weights. Moreover, as in soleus muscle, we again note the absence of a non-phosphorylated species in FDB muscle. This may suggest that sMyBP-C is constitutively phosphorylated at basal levels. Conversely, it may be the result of coincident migration of non-phosphorylated higher molecular weight and singly or doubly phosphorylated lower molecular weight variants.

Compared to young wild type FDB muscle, the expression and relative abundance of select species varies significantly in aged or dystrophic FDB muscles (Fig. 5a). Accordingly, the low mobility species denoted with a red dot is only expressed in aged wild type FDB muscle where it exhibits a relatively low abundance (~10%). Notably, the intermediate mobility species marked with a bright yellow dot is expressed in aged wild type and young, but not old, *mdx* FDB muscles where it displays a relatively low expression (~5% and ~6%, respectively). Moreover, the relative abundance of the high mobility species marked with a pink dot varies significantly among the four FDB muscle groups, ranging from ~21% in young wild type to 5% in young *mdx* tissue. In addition, the relative expression of the intermediate mobility species marked with a brown dot increases significantly in aged wild type (~10%) and *mdx* (~12%) FDB samples compared to young wild type tissue (~4%), but decreases dramatically (~1%) in young *mdx* tissue. On the contrary, the levels of the low (yellow dot) and intermediate (dark green and dark blue dots) mobility species remain relatively constant among the different FDB muscle groups (34–41%, 20–33% and 4–5%, respectively). Lastly, the intermediate mobility species marked with turquoise and purple dots exhibit low, yet similar, relative expression in all muscle groups (~3% and ~5%, respectively), with the following exceptions: i. the relative abundance of the former increases in young *mdx* FDB muscle (~9%), and ii. the latter is absent from aged wild type FDB muscle.

### The phosphorylation profile of sMyBP-C in human abductor hallucis and gastrocnemius muscles from DA-1 patients

Genome wide linkage analysis has revealed that mutations in *MYBPC1* encoding sMyBP-C lead to the development of Distal Arthrogryposis type-1 (DA-1)^12^. To date, DA-1 is the most common among the ~10 identified forms of distal arthrogryposis myopathy^13^. Two missense mutations in *MYBPC1*, W236R and Y856H residing in the M-motif and the C8 domain, respectively, have been linked to the development of DA-1^12^. We have begun to assess the effect of each of these mutations on the phosphorylation profile of sMyBP-C. To this end, we used standard and phosphate affinity gel electrophoresis in combination with a panel of phospho-specific antibodies to examine the effects of the DA-1 mutations on the phosphorylation profile of sMyBP-C in distal and proximal muscles. Specifically, we used human biopsies of AH, a distal foot muscle carrying the Y856H mutation, and gastrocnemius, a proximal leg muscle containing the W236R mutation. Gastrocnemius muscle carrying the W236R mutation contains nearly identical levels of total and phosphorylated sMyBP-C protein compared to control tissue. However, AH muscle carrying the Y856H mutation exhibits significantly reduced levels of total sMyBP-C protein (~25%) and of phosphorylation at each of the four phospho-sites (~32–70%), compared to control tissue (Fig. 6). More importantly, the presence and relative abundance of individual phosphorylation events or combinations thereof vary considerably in AH muscle carrying the Y856H mutation, but not in gastrocnemius muscle containing the W236R mutation (Fig. 6a,b). These findings indicate that the phosphorylation profile of sMyBP-C is altered in pathologically affected distal muscles but not in unaffected proximal muscles in DA-1 myopathy.

**Figure 9:**
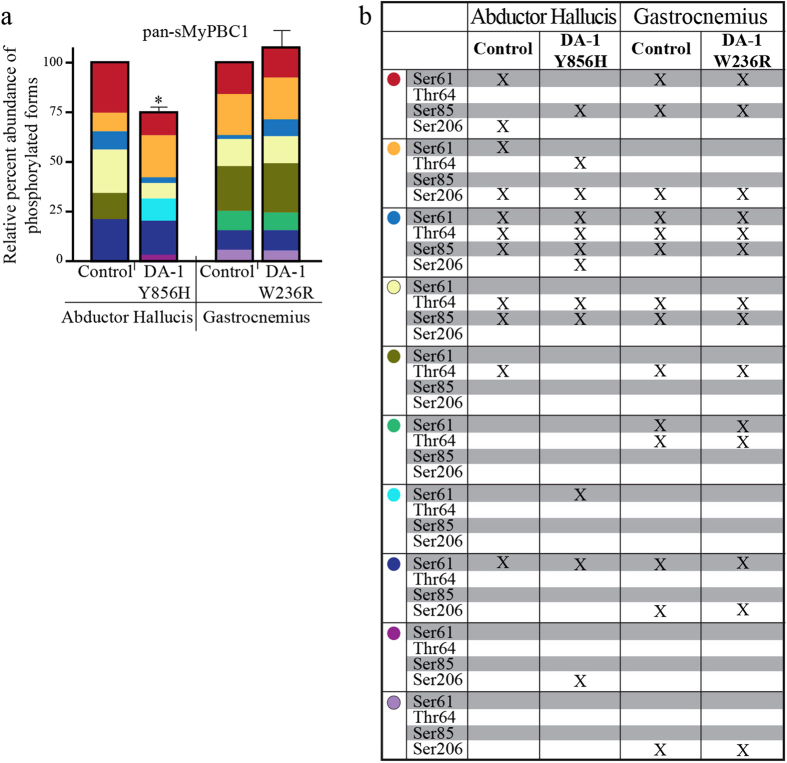
Relative abundance and phosphorylation profile of sMyBP-C forms present in AH and gastrocnemius muscles from human patients carrying the DA-1 mutations. (**a**) The relative abundance of each immunoreactive band detected with the α-pan-sMyBP-C antibody following separation of protein lysates by phosphate affinity SDS-PAGE (Fig. 6b) was calculated as percent expression of the total protein content within each sample. Significance is as described in Fig. 6a’. (**b**) The phosphorylation events detected in each sMyBP-C form within the different samples were determined by comparative evaluation of the phosphate affinity SDS-PAGE immunoblots shown in Fig. 8. The colored dots correspond to sMyBP-C immunoreactive bands with specific electrophoretic mobility as determined by phosphate affinity SDS-PAGE, and thus may represent different (i.e. bands denoted with red, yellow, tan, and dark blue dots) or the same (bands denoted with light yellow, olive green, light green, turquoise, purple, and light purple dots) phospho-species across the human samples.

Similar to soleus and FDB muscles, we again performed a comparative analysis of the results shown in Figs 6, 7, 8 to assess the relative abundance (Fig. 9a) and phosphorylation events present in individual sMyBP-C species (Fig. 9b) in control and DA-1 muscles. Of the six sMyBP-C forms expressed in control AH tissue, there are three highly abundant forms, one of low (red dot, ~27%) and two of intermediate (light yellow and dark blue dots; ~24% and 19%, respectively) mobility. Interestingly, no immunoreactive band is phosphorylated at all four known sites in control AH muscle. With the exception of a low mobility species (blue dot) that is of low relative abundance (<10%) and phosphorylated at all three sites present in the Pro/Ala rich motif, the remaining species are either singly or doubly phosphorylated at different combinations of residues.

Compared to control AH muscle, DA-1 AH muscle carrying the Y856H mutation exhibits a more complex phosphorylation profile (Fig. 9a). The most prominent species in DA-1 AH is a low mobility form (yellow dot), which exhibits a considerable increase (~30%), while three species, two of low (red and blue dots) and one of intermediate (light yellow dot) mobility exhibit lower relative abundance (~15%, ~4%, and ~10%, respectively), compared to control tissue. Interestingly, three of the five species shared between control and DA-1 AH tissues (red, yellow, and blue dots) exhibit different phosphorylation profiles. Specifically, the low mobility species denoted by a red dot is doubly phosphorylated at hSer61 and hSer206 in control AH tissue, but singly phosphorylated at hSer85 in DA-1 AH muscle. Similarly, the low mobility species marked with a blue dot is phosphorylated at all four known residues in DA-1 AH, opposite to control AH where it is phosphorylated only at the three sites present within the Pro/Ala rich motif. These findings suggest that these species either correspond to sMyBP-C variants of different molecular weights and phosphorylation profiles that exhibit similar electrophoretic mobility, or to the same variant that is differentially phosphorylated between muscles, at known and/or unknown residues.

Similar analysis of control and DA-1 gastrocnemius muscle carrying the W236R mutation revealed no significant differences in the relative abundance or phosphorylation profile of individual sMyBP-C species.

Taken together, our findings show that the distal AH muscle carrying the Y856H mutation exhibits altered expression and phosphorylation of sMyBP-C, while the proximal gastrocnemius muscle carrying the W236R mutation does not. It is possible that these differential effects in distal and proximal muscles may arise from the individual mutations. We find this highly unlikely though, given that previous studies have demonstrated that DA-1 AH muscles carrying either the W236R or the Y856H mutation exhibit similar pathologies, as evidenced by the presence of smaller type-I fibers compared to matching controls^12^. Thus, it is possible that the presence of the W236R mutation in AH muscle may also result in major alterations of the expression levels and the phosphorylation profile of sMyBP-C. Conversely, the presence of the Y856H mutation in gastrocnemius muscle may not have any effect in either its expression levels or its phosphorylation profile.

An important question that still remains, however, is how the Y856H mutation residing in Ig C8 at the COOH-terminus of the molecule affects its phosphorylation pattern within its NH_2_-terminus. It has been previously shown both *in vitro* and *in vivo* that the introduction of either DA-1 mutation does not alter the normal distribution of sMyBP-C to the sarcomeric A-band^12^,^24^,^25^. However, it is possible that the Y856H mutation may disrupt the proper orientation of sMyBP-C within the sarcomere, its conformation or its tertiary structure, which in turn may result in reduced accessibility of its NH_2_-terminus to PKA and PKC. High-resolution cellular and structural studies may provide answers to this important question and help us discern the underlying molecular mechanisms that lead to altered phosphorylation of sMyBP-C in DA-1 myopathy.

### Concluding remarks

Using the fast-twitch mouse FDB muscle and the human AH and gastrocnemius muscles as model systems, we evaluated the phosphorylation profile of sMyBP-C in normalcy and under different pathophysiological conditions. Our studies are the first to demonstrate that the phosphorylation pattern of the sMyBP-C sub-family is differentially regulated in response to age and disease. Future studies will focus on the specific roles that each phosphorylation event or combinations thereof play on the functional properties of sMyBP-C proteins.

## Methods

### Animal Models and Tissue Collection

All animal protocols were conducted in accordance with the approved protocols of the Institutional Animal Care and Use Committee of the University of Maryland School of Medicine. FDB muscles were obtained from young (~2 months) and old (~14 months) adult male wild type C57BL/6Scsn/J and dystrophic *mdx* C57BL/10Scsn-Dmd mdx/J mice (Jackson Laboratories, Bar Harbor, ME). Immediately following isolation, tissues were snap-frozen in liquid nitrogen. Four (n = 4) FDB muscles were collected from different mice.

### Human Tissue Samples

Human muscle biopsies of AH and gastrocnemius muscles carrying the DA-1 Y856H and W236R mutations, respectively, were obtained during reconstruction surgery; both patients exhibited similar levels of disease severity. Control AH and gastrocnemius muscles that do not contain the DA-1 mutations were obtained from 15- and 20-months old patients with clubfoot and a 16-year old trauma patient with no myopathy, respectively. Tissue samples were flash-frozen in liquid nitrogen. Moreover, all tissue samples were collected in accordance with a protocol approved by the Institutional Review Board of the Washington University School of Medicine, and with the consent of all subjects. The available information for each human sample is noted in Table 1.

### Generation of Protein Lysates and Western Blotting

Protein lysates from mouse FDB muscles were generated as previously described^1^. Lysates (~30 μg) from each tissue were prepared for electrophoresis and heated at 90 °C for 5 minutes. Separation of proteins was conducted using either conventional SDS-PAGE (Life Technologies, Carlsbad, CA)^10^,^11^, or phosphate affinity SDS-PAGE, according to the manufacturer’s instructions. Separation via phosphate affinity SDS-PAGE was performed using 100 μmol/L Phos-tag^TM^ (Wako Chemicals USA, Inc. Richmond, VA), and a 10% w/v solution of acrylamide^26^. Following electrophoretic separation, lysates were subjected to western blot analysis and probed with the indicated antibodies. A pan-sMyBP-C antibody (mouse monoclonal; 300 ng/ml; Abnova, Walnut, CA) and an Hsp90 antibody (mouse monoclonal; 300 ng/ml; Cell Signaling Technology, Inc., Danvers MA) were used per the manufacturers’ instructions. Evaluation of the entire lanes following immunoprobing with the pan-sMyBP-C antibody indicated the absence of any (detectable) degradation of sMyBP-C in the mouse and human muscle lysates (SFig. 1). In addition, phospho-specific custom polyclonal antibodies to each of the phospho-sites of sMyBP-C, mSer-59/hSer-61, mSer-62/hThr-64, mThr-84/hSer-85, and mSer-204/hSer-206 were used (750 ng/ml, Ackermann *et al.* 2014; Companion paper). Antibody validation and specificity are described in detail in (Ackermann *et al.* 2014; Companion paper).

### Statistical Analysis

Using standard SDS-PAGE blots and ImageJ software (NIH, Bethesda, MD), we quantified the relative content of total sMyBP-C and the relative expression of each phosphorylated residue within a sample. Percent (%) expression was normalized to the loading control, Hsp90, and subsequently to the levels of sMyBP-C present in the mouse young wild type FDB or the appropriate human control clubfoot muscle. Significance was calculated via student’s t-test (p < 0.01). Moreover, we quantified the relative abundance of individual immuno-reactive bands within a sample using Image J software. To this end, the quantified relative content of total sMyBP-C for each sample calculated above, served as a representation of total sMyBP-C protein within that sample. The relative abundance of each immuno-reactive band identified in the phosphate affinity SDS-PAGE blots by the pan-sMyBP-C antibody was calculated as a percentage of the total protein within that sample. Furthermore, we used the phosphate affinity SDS-PAGE western blots probed with each of the phospho-specific antibodies to ascertain the phosphorylation events within a specific immuno-reactive band detected with the pan-sMyBP-C antibody.

## Additional Information

**How to cite this article**: Ackermann, M. A. *et al.* Myosin Binding Protein-C Slow Phosphorylation is Altered in Duchenne Dystrophy and Arthrogryposis Myopathy in Fast-Twitch Skeletal Muscles. *Sci. Rep.* **5**, 13235; doi: 10.1038/srep13235 (2015).

## Figures and Tables

**Figure 1:**
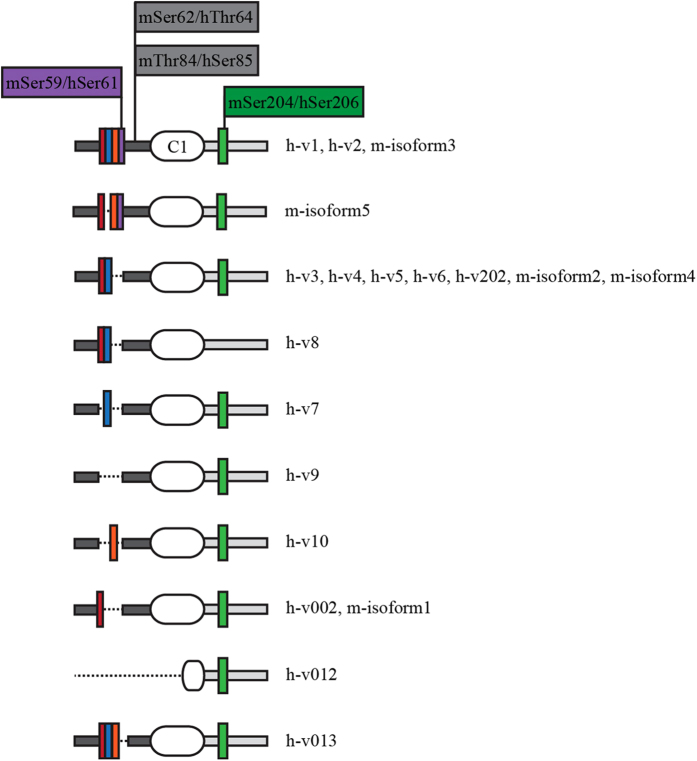
The NH_2_-terminus of sMyBP-C is subjected to phosphorylation. Schematic representation of the NH_2_-terminus of the known human and mouse sMyBP-C variants with the phosphorylation sites highlighted. The Pro/Ala rich and M- motifs are denoted as dark and light grey rectangles, respectively, and the first Ig domain (C1) is shown as a white oval. Colored rectangles indicate short stretches of amino acids that are products of alternatively spliced regions.

**Table 1:** Control and DA-1 mutant human tissue samples.

Patient	Mutation	Gender	Age at biopsy	Tissue	Diagnosis
5432001	W236R	M	5 yr	Gastrocnemius	Distal arthrogryposis-1
8039001	Y856H	M	3 yr	Abductor Hallucis	Distal arthrogryposis-1
2017	None	M	16 yr	Gastrocnemius	Trauma/No myopathy
5263004	Unknown	M	15 mos	Abductor Hallucis	Bilateral clubfoot, familial
5476001	Unknown	M	20 mos	Abductor Hallucis	Left clubfoot, familial
